# Depth-Dependent Spatiotemporal Dynamics of Overwintering Pelagic *Microcystis* in a Temperate Water Body

**DOI:** 10.3390/microorganisms9081718

**Published:** 2021-08-12

**Authors:** Haolun Tian, Junjie Jin, Bojian Chen, Daniel D. Lefebvre, Stephen C. Lougheed, Yuxiang Wang

**Affiliations:** 1 Department of Biology, Queen’s University, Kingston, ON K7L 3N6, Canada; allen.tian@queensu.ca (H.T.); 15jj14@queensu.ca (J.J.); lefebvre@queensu.ca (D.D.L.); lough@queensu.ca (S.C.L.); 2 College of Environmental Science and Engineering, Tongji University, Shanghai 200092, China; chenbojian@tongji.edu.cn

**Keywords:** algal bloom, CHAB, vertical distribution, cyanobacteria, microcystin, real-time PCR

## Abstract

Cyanobacteria in the genus *Microcystis* are dominant components of many harmful algal blooms worldwide. Their pelagic–benthic life cycle helps them survive periods of adverse conditions and contributes greatly to their ecological success. Many studies on *Microcystis* overwintering have focused on benthic colonies and suggest that sediment serves as the major inoculum for subsequent summer blooms. However, the contemporaneous overwintering pelagic population may be important as well but is understudied. In this study, we investigated near-surface and near-bottom pelagic population dynamics of both microcystin-producing *Microcystis* and total *Microcystis* over six weeks in winter at Dog Lake (South Frontenac, ON, Canada). We quantified relative *Microcystis* concentrations using real-time PCR. Our results showed that the spatiotemporal distribution of overwintering pelagic *Microcystis* was depth dependent. The abundance of near-bottom pelagic *Microcystis* declined with increased depth with no influence of depth on near-surface *Microcystis* abundance. In the shallow region of the lake (<10 m), most pelagic *Microcystis* was found near the lake bottom (>90%). However, the proportion of near-surface *Microcystis* rose sharply to over 60% as the depth increased to approximately 18 m. The depth-dependent distribution pattern was found to be similar in both microcystin-producing *Microcystis* and total *Microcystis.* Our results suggest the top of the water column may be a more significant contributor of *Microcystis* recruitment inoculum than previously thought and merits more attention in early CHAB characterization and remediation.

## 1. Introduction

Cyanobacterial harmful algal blooms (CHABs) are an increasingly common consequence of eutrophication in freshwater systems. Typically defined as a rapid increase in localized cyanobacterial biomass [1,2], CHABs can have significant negative impacts on aquatic ecosystems, reducing water quality [3], creating asphyxiating hypoxic ‘dead zones’ [4], limit nutrient turnover [5], and producing potent cyanotoxins [6,7,8]. Their impact is increasing worldwide, promoted by nutrient loading [9,10], rising atmospheric CO_2_ concentrations [11] and rising temperatures [12,13,14,15]. Current strategies in CHAB control typically include nutrient-loading reduction and post-bloom control [16]. However, this has proved ineffective despite large investments in the past two decades, for example in Lake Taihu in Eastern China [17] and in Lake Erie in North America [18]. Reducing phosphorus and nitrogen inputs alone can be ineffective due to diverse and global causes of proliferation, such as rising atmospheric CO_2_ [19,20]. Additionally, reducing inputs may not be feasible in developing countries due to population pressures and limited budgets. Effective and economical control of CHABs requires a proactive approach with a focus on targeted remediation of nascent blooms during their formation [21]. This requires extensive knowledge of the natural history, mechanisms, and distributions of CHAB-forming cyanobacterial species.

The genus *Microcystis* is a major cyanotoxin produced in temperate freshwater CHABs [22]. The life history of *Microcystis* and other coccoid colony-forming cyanobacteria consists of a benthic, overwinter resting phase, a pelagic, planktonic phase, and a colonial, blooming phase [23,24,25,26]. The main sources of inoculum for growth and recruitment in spring are thought to be concentrated in the lake bottom and sediment during winter, originating from *Microcystis* colonies that settle in the water column in the fall after summer blooms [27]. There, most of the population die while the survivors enter a dormant resting phase during which little photosynthesis or respiration occurs [27,28]. The spatial distribution of overwintering *Microcystis* seed stock in the water column across different depths is not well understood despite their high overwintering viability [27,29,30]. While water–sedimentary sources are thought to be the dominant source of bloom inoculum, modeling indicates that the smaller overwintering pelagic population is a greater predictor of the magnitude of summer blooms than the near-bottom population, implying that pelagic *Microcystis* is potentially more significant as a source of inoculum than their biomass suggests [29]. Overwintering survival is favored by low light availability and low dissolved oxygen in laboratory conditions [27]. Although this may seem to favor near-bottom *Microcystis* present in deeper areas of a water body, ice and snow cover restricts light availability in all areas, and reduced photosynthetic activity, surface oxygenation, and water flow during the winter means that low dissolved oxygen conditions are probably similarly uniform. Wind-induced mixing and bioturbation after ice melt may have a greater role in the resuspension of overwintering *Microcystis* in the near-bottom and sediment than active buoyancy control [30]. It is likely that there is greater recruitment success in shallow lakebed areas, which experience greater mixing than deeper areas [31]. Stratified lakes also have a littoral benthic zone that overlaps with the mixed epilimnion-hypolimnion interface, while the profundal benthic zone is typically physically separated from surface–water interactions during the stratified periods that make up the majority of their annual cycle [32]. Therefore, viable overwintering *Microcystis* populations in the near-bottom may be higher in areas with shallower lakebeds and lower in deeper, aphotic areas of a lake due to weaker passive recruitment processes and a lack of interaction with the photic zone. In these deeper areas of the lake, the uppermost pelagic *Microcystis* may be a more significant source of inoculum and have a higher relative population. Understanding this overwintering distribution of *Microcystis aeruginosa* has important implications for early bloom prediction and bioremediation, as overwintering survival is likely to be strongly correlated with early spring recruitment, biomass build up, and the formation of nascent cyanobacterial blooms.

An additional potential source of inoculum that has rarely been considered in temperate lakes is ice cover. Although viable *Microcystis viridis* has been isolated and identified from frozen pieces of ice cover [32], the potential of ice cover as a general source of cyanobacterial inoculum has yet to be explored in a natural, stratified lake and relative to other sources. Ice cover may be an important overwintering source of inoculum, particularly as the viability of frozen *Microcystis viridis* colonies on agar plates was much higher than samples from the water column or sediment in Vasas’ study [32]. Freeze–thaw viability of cyanobacteria in lab conditions is typically high [33,34], and it is possible that planktonic *Microcystis* in the form of small colonies or single cells may also survive overwintering in the ice cover as a potential inoculum source in general. Therefore, we hypothesized that ice cover contains viable *Microcystis aeruginosa*, although in smaller quantities than in the water column.

Factors that influence the toxicity of CHABs are not well understood. It is well established that cyanobacteria within a lake can exhibit high genetic diversity [35], and toxigenic potential may follow clear spatial patterns [36,37]. However, differences in overwintering distribution between toxic and non-toxic strains have yet to be explored. Toxicity in cyanobacteria has been linked to planktivory, high temperatures, and UV damage. As zooplankton and solar irradiation are more prevalent in the littoral regions of lakes, toxicity may decrease in near-bottom cyanobacteria as the depth increases.

Environmental DNA (eDNA) is an emerging approach to biomonitoring that allows us to take a quantitative biodiversity snapshot of an ecosystem. Organisms leave traces of their genetic material as they move through their environment [38], often in the form of intact cells [39]. In the case of microbial organisms such as cyanobacteria, sampling involves collecting the entire organism. After DNA extraction, molecular biology techniques such as quantitative PCR (qPCR) can be used to detect and even quantify specific species or genes of interest [40,41]. In the case of barcoding genes such as the 16S rRNA gene, qPCR can be used to quantify relative species abundance [42]. In *M. aeruginosa*, the 16S rRNA and *mcyE* genes have long been used to detect and quantify populations of total and toxic strains [43,44,45]. Through consideration of filter pore size, material, and flow rate, eDNA capture can be limited to intact cells [39].

Our study used qPCR on the *M. aeruginosa* 16S rRNA gene to compare the relative abundance of overwintering pelagic populations in the near-surface, near-bottom, and ice cover at sites of a temperate lake with different lakebed depth. We hypothesized that near-surface *Microcystis* is most abundant in shallow areas, while near-bottom *Microcystis* is more prevalent in deeper areas of the lake due to later ice cover formation and a more stable water column. Finally, ice samples have a viable population of *Microcystis*. Overall distribution of *Microcystis* should favor shallow areas due to greater recruitment success. We also quantified the relative proportion of toxic strains by comparing the *M. aeruginosa mcyE* and 16S rRNA genes. We hypothesized that the toxigenic potential of near-bottom overwintering *Microcystis* populations within a lake decreases as depth increases.

## 2. Materials and Methods

### 2.1. Study Site

**Figure 1:**
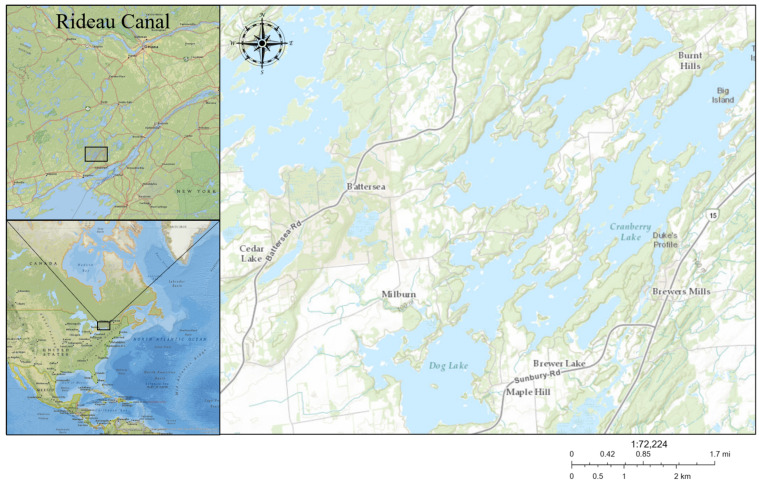
Map of Dog Lake, South Frontenac County, ON, Canada. The Rideau Canal watershed is shown in the top left while Eastern North America is shown in the bottom left. Dog Lake’s center basin is located at 44.435449° N, 76.345772° W.

Our study site was at Gilmour Point (44.432107° N, 76.354664° W), a small peninsula with a public beach and boat launches at Dog Lake, South Frontenac County, ON, Canada (Figure 1). Gilmour Point is in the middle basin of Dog Lake, which is a eutrophic lake on the Rideau Canal system primarily used for agricultural, vacation residences, and recreational boating activity. This site was ideal for our study due to the large variation in depth, historic cyanobacterial presence, thick ice cover, and easy road access. Transects from Gilmour Point beach to the middle of the basin have a range of maximum depths, from 0 to ~25 m. Gilmour Point has a history of regular annual CHABs, with a large bloom observed in the summer of 2018, which provided an excellent opportunity for sampling.

### 2.2. Sampling Scheme

We conducted weekly sampling from 7 February to 12 March of 2019, collecting a total of 153 water samples across 6 sampling days. We collected 69 of those samples from the top 0.5 m of the water column, which we designated as near-surface samples, 69 samples from the bottom 0.5 m of the water column, which we designated as near-bottom samples, and 15 samples from the ice cover (Supplementary Table S1). We also collected time/date and depth data for each sampling point.

**Figure 2:**
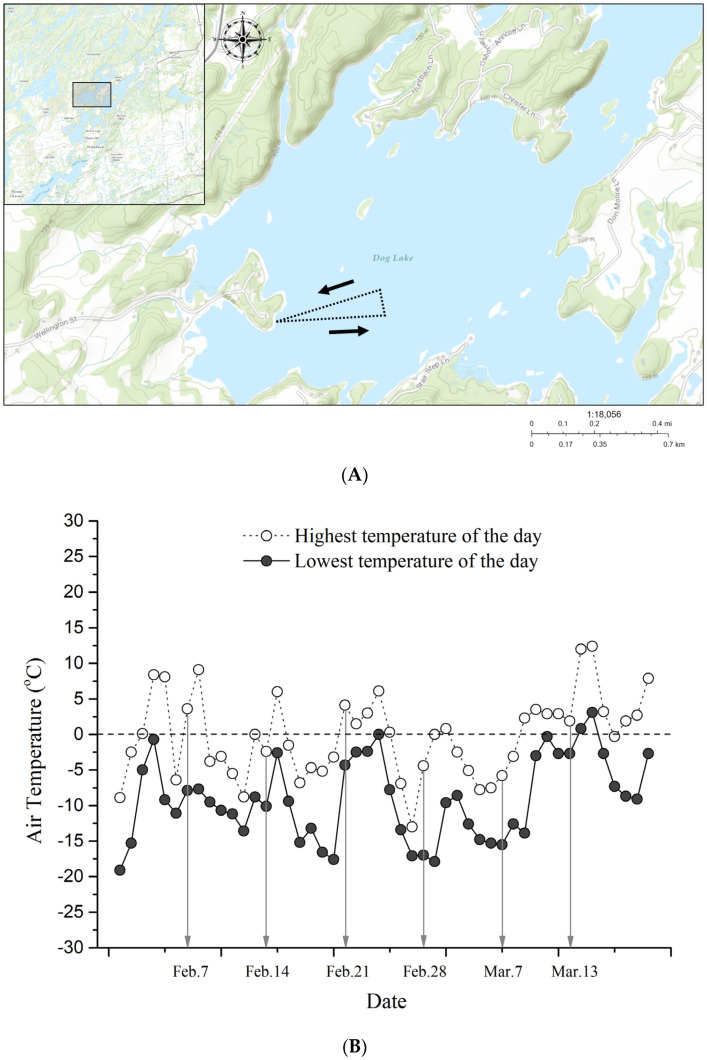
Site sampling information. (**A**) Site map of Gilmour Point, Dog Lake, South Frontenac County, Ontario (44.435635° N, 76.345549° W). Sampling occurred along two transects, with each site roughly equidistant from the origin point with 5–8 samples collected per transect (Supplementary Table S1). (**B**) Ambient temperature at each sampling time. The arrows point at sampling dates. Data were obtained from a Acurite 02064M Pro weather station (Chaney Instrument Co, Lake Geneva, WI, USA) set up in accordance with manufacturer’s specifications at Maple Hill, South Frontenac County, Ontario, Canada (44.398594° N, 76.352457° W).

We sampled across two transects, originating at 44.432104° N, 76.354408° W, and extending approximately 300 m from the beach towards the middle of the basin (Figure 2). After collecting samples at half of our sampling sites, we travelled approximately 100 m north and collected the remaining samples in a second transect towards Gilmour Point beach. Although sampling in a randomized grid would have eliminated potential order effects, navigating the frozen lake surface on foot made this difficult. These two transects covered a range of depths, from 1 to 18 m. Sampling points were located at least 80 m apart, and we avoided sampling within 10 m of a previous sampling location, as indicated by auger holes, whenever possible to maximize spatial coverage. As dimictic lakes are typically vertically stagnant during the winter, and our study area was not in the main flow of the Rideau Canal, we assumed that proximity effects were minimal. We drilled a hole through the ice using an 8-inch gas-powered ice auger (Eskimo, Cumberland, WI, USA) and measured the depth using a weighted 0.5-m graduated rope. For the ice cover sample collection, we paused drilling before reaching the water, and collected 1000 mL of packed ice fragments using plastic jars. For water sample collection, we finished drilling through the ice cover to the water column and removed any ice and snow fragments from the water using a sieve. We then collected surface water samples by dipping a sterile plastic jar into the water with a reach-grabber tool. We collected bottom water samples from within 0.5 m of the lake bottom using a 1000-mL double flap valve acrylic bailer sampler (unbranded, from AliExpress) with an attached extension which prevented it from reaching the lakebed and disturbing the sediment. We stored water samples in 500-mL clear wide-mouth PET plastic jars, which were kept covered from light in a chilled 48 QT Coleman cooler (Coleman Company, Inc., Chicago, IL, USA) during transport to the lab and filtered them for eDNA within 3 h of collection. We sterilized jars with hot water and soap, and immersion in 10% bleach for 24 h between uses.

### 2.3. Sample Filtration

Our filtration protocol was adapted from Turner et al. [39] and Feng et al. [46]. We filtered water samples for environmental DNA using IsoporeTM polycarbonate tract-etched (PCTE) membrane filters (1.2-μm pore size, 47-mm diameter, from Millipore-Sigma, Oakville, ON, Canada), housed in a 47-mm in-line filter holder (Pall Corporation, Port Washington, NY, USA) and passed through with a portable peristaltic pump (Wattera, Mississauga, ON, Canada). We passed 250 to 400 mL of each water sample, depending on the turbidity, through each of the PCTE membrane filters. We recorded the volume filtered for each sample. As *Microcystis* cells range from 2–8 μm in diameter, this protocol captures all intact cells, but the filtration rate and pore size minimize DNA adsorption to the PCTE membrane filter. Therefore, eDNA captured by this technique should mostly originate from live, viable cyanobacteria. After filtration, we folded the filters with flame sterilized forceps and stored them in 500 μL of 2% (*w/v*) cetrimonium bromide extraction buffer (CTAB) in 2-mL conical microcentrifuge tubes (Bio Basic Inc., Markham, ON, Canada). We stored all filters in a −20 °C freezer until DNA extraction.

### 2.4. DNA Extraction

We extracted DNA using a chloroform/ethanol-based method modified from Deiner and Altermatt [47]. We initially incubated the PTCE filters in CTAB at 65 °C for 10 min. Following this, we added an equal volume of chloroform–isoamyl alcohol (24:1 ratio), shook each tube until the filter fully dissolved, and centrifuged at 15,000 g for 15 min. We then transferred the aqueous phase with a p1000 pipette to a new 1.7-mL graduated microcentrifuge tube (Lifegene, Mevo Horon, Israel) and added an equal volume of ice-cold isopropanol and half the volume of 5 M NaCl. We gently mixed and then incubated these at room temperature for a minimum of 1 h, and then centrifuged at 15,000 g RCF for 15 min. We decanted and discarded the supernatant, added 150 μL of ice cold 70% ethanol, and centrifuged again at 15,000 g RCF for 15 min. We decanted the ethanol and repeated this wash-step once. We then left the resulting pellet to air dry for 30 min. We resuspended the pellet in 50 μL of 65 °C 1× AE buffer (Qiagen, Saint-Catherine, Montreal, QC, Canada). We stored DNA samples in a −20 °C freezer.

### 2.5. Primer Design and Standards

We made standards for qPCR using two strains of *M. aeruginosa,* CPCC 124 and CPCC 300, purchased from the Canadian Phycological Culture Centre (Waterloo, ON, Canada). CPCC 124 is a non-toxic strain isolated by J. Acreman in July of 1987 from Heart Lake, Ontario, Canada, while CPCC 300 is a toxic strain (producing 204 μg_microcystin_ g^−1^ of dry weight) isolated by A. Lam from Pretzlaff Pond, Alberta, Canada [48].

We maintained axenic cultures of *M. aeruginosa* in 80 mL of sterile cyanobacteria growth medium adapted from Ichimura [49] in 120 mL borosilicate culturing tubes. Growth medium contained NaNO_3_ (50 mg L^−1^); KNO_3_ (100 mg L^−1^); Ca (NO_3_)_2_∙4H_2_O (50 mg L^−1^); Na_2_SO_4_ (40 mg L^−1^); MgCl_2_∙6H_2_O (50 mg L^−1^); Na_2_ b-glycerophosphate∙5H_2_O (50 mg L^−1^); Na_2_-EDTA (5 mg L^−1^); FeCl_3_∙6H_2_O (0.5 mg L^−1^); MnCl_2_∙4H_2_O (5 mg L^−1^); ZnCl_2_ (0.5 mg L^−1^); CoCl_2_∙6H_2_O (5 mg L^−1^); Na_2_MoO_4_∙2H_2_O (0.8 mg L^−1^); H_3_BO_3_ (20 mg L^−1^); and C_6_H_13_NO_4_ (500 mg L^−1^), with the pH adjusted to 8.6 with aqueous NaOH. We kept cultures under a 16/8 H day/night cycle at 400 μmol m^−2^ s^−1^. We replenished the growth medium monthly and examined cultures under microscopy for contamination.

We extracted genomic DNA from the two reference stains using a DNeasy^®^ Mini Plant kit (Qiagen, Saint-Catherine, Canada) following the manufacturer’s instructions. We obtained a rough estimate of DNA quality and quantity using a Nanodrop ND1000 spectrophotometer (Thermo Fisher Scientific Inc., Waltham, MA, USA) and amplicon size with gel electrophoresis and a 100-bp ladder (New England Biolabs, Ipswich, MA, USA) on a 1.5% agarose gel stained with RedSafe^®^ (FroggaBio Inc., Concord, ON, Canada). We stored extracted DNA in a −20 °C freezer.

We tested the primers from Table 1 for specificity on the axenic *Microcystis* cultures, with axenic cultures of *Anabaena*, *Chlorella*, and *Oscillatoria* as negative controls. PCR with the *Microcystis* 16S rRNA gene primers on genomic DNA extracted from the *Microcystis* strain CPCC 124 yielded an amplicon of 220 bp, and PCR with the *Microcystis mcyE* gene primers yielded an amplicon of 120 bp. PCR with both primer sets on genomic DNA from *Anabaena*, *Chlorella*, and *Oscillatoria* yielded no amplicons. This confirmed specificity.

We determined the melting temperatures of the two primer sets with a CFX96 TouchTM Real-Time PCR Detection System (Bio-Rad, Hercules, CA, USA) through raising the temperature from 65 to 95 °C and quantifying the fluorescence in this range. *Microcystis* 16S rRNA gene primers produced peak fluorescence at a qPCR melting temperature of 86 °C, while *Microcystis mcyE* gene primers produced peak fluorescence at a qPCR melting temperature of 78 °C. There was no evidence of primer dimers.

To produce standards for our two gene fragments, we performed PCR using a GeneAmp^®^ PCR System 9700 (Thermo Fisher Scientific Inc., Waltham, MA, USA) on genomic DNA previously extracted from the two *Microcystis* reference strains. The PCR reactions contained 10 μL of 2× Taq FroggaMix (FroggaBio Inc., Toronto, ON, Canada), 2 μL of extracted genomic DNA, 0.8 μL of both forward and reverse primers (to a final concentration of 0.3 μM in the final 20 μL reaction), 10 μg of bovine serum albumin (Life Technologies Corporation, Carlsbad, CA, USA), and 5.9 μL of ddH_2_O. Our PCR amplification protocol was as follows: initial denaturation of 5 min at 98 °C, 40 cycles of denaturation at 98 °C for 30 s, annealing at 60 °C for 30 s, and extension at 72 °C for 60 s, a final extension at 72 °C for 10 min, and pause at 4 °C. We confirmed the presence of the PCR product through gel electrophoresis on a 1.5% agarose gel stained with RedSafe^®^ nucleic acid staining solution. We extracted DNA from the gel with a QIAquick Gel Etraction Kit (Qiagen, Hilden, Germany) and quantified the resulting product using a DeNovix dsDNA High Sensitivity Kit on a DeNovix QFX Fluorometer (DeNovix Inc., Wilmington, NC, USA). We then calculated the molecular weights of the PCR products with the following equation, where M is the mass of amplicon in ng µL^−1^, BP is the length of the amplicon in base pairs, and 660 g mol^−1^ is the average molar mass of 1 base pair of double stranded DNA:

Copies=M×(6.0221409×1023moleculesmole)(BP×660gmole)×(1×109ngg)

We performed seven 10-fold serial dilutions of the purified PCR product to generate standard curves for the *Microcystis* 16S rRNA and 16S gene fragments. qPCR on the standards yielded an *R*^2^ value of over 0.998 between the log_10_ of the gene copies and threshold cycle (CT) values, indicating that the standards were valid and accurate.

### 2.6. Quantitative PCR

We detected the quantity of total and potentially toxigenic *M. aeruginosa* through performing a SYBR Green-based qPCR assay for the previously mentioned *Microcystis* 16S rRNA gene fragment and *mcyE* gene fragment on our field samples. We performed qPCR reactions in a total volume of 20 μL, containing 10 μL 2× SensiFAST SYBR Green Master Mix (FroggaBio, Toronto, ON, Canada), 2 μL of DNA from field samples or standards, 0.8 μL of each primer (to a final concentration of 0.3 μM in the final 20 μL reaction), 0.5 μL (containing 10 μg) of bovine serum albumin, and 5.9 μL of ddH_2_O. We plated reactions in triplicate on clear 96-well PCR plates (FroggaBio, Toronto, ON, Canada) and used a CFX96 TouchTM Real-Time PCR Detection System. The qPCR amplification protocol for both the 16S rRNA gene fragment and *mcyE* gene fragment is as follows: initial denaturation of 3 min at 95 °C, 40 cycles of 5 s of denaturation at 95 °C, and 15 s of annealing/extension at 57 °C. We corrected qPCR gene copy concentrations by the volume filtered for each sample.

### 2.7. Statistics

We conducted statistical analyses using IBM SPSS Statistics 26 (IBM, Armonk, NY, USA), and generated all maps in ArcGIS (Esri, Redland, CA, USA). We tested for normality of error distributions using Kolmogorov–Smirnov tests and log_10_ transformed concentration data of *M. aeruginosa* 16S rRNA and *mcyE* gene copies prior to the statistical analyses.

We conducted an ANOVA, followed by Tukey’s HSD post hoc testing to test for significant differences in gene concentrations between samples collected from the near-surface, the near-bottom, and ice cover.

To determine whether the vertical distribution of overwintering *Microcystis* population was influenced by water column level (top, bottom), we applied univariate general linear models (GLM) using “sampling time” and “water column level” as factors. “Depth” (depth of each sampling site) was included as covariate, and concentrations of *M. aeruginosa* gene copies (16S rRNA or *mcyE*) were used as the dependent variable. All two-way interactions were initially included but were excluded from the final models if non-significant (*p* > 0.05). To test for the correlation between *Microcystis* concentration and lake depth in different parts of the water column, we created linear regressions between log_10_ transformed *mcyE* and 16S rRNA gene concentrations with lake depth. We did this separately for near-surface and near-bottom samples.

We tested the ratio of the toxigenic *mcyE* gene to the general 16S rRNA gene as a proxy for the ratio of potentially toxigenic *Microcystis* to overall *Microcystis*. We conducted linear regressions between the ratio of the log_10_ transformed *mcyE* to 16S rRNA gene concentrations with depth.

For all regressions, we assessed heteroscedasticity and linearity through graphical assessment of residual statistics.

## 3. Results

**Figure 3:**
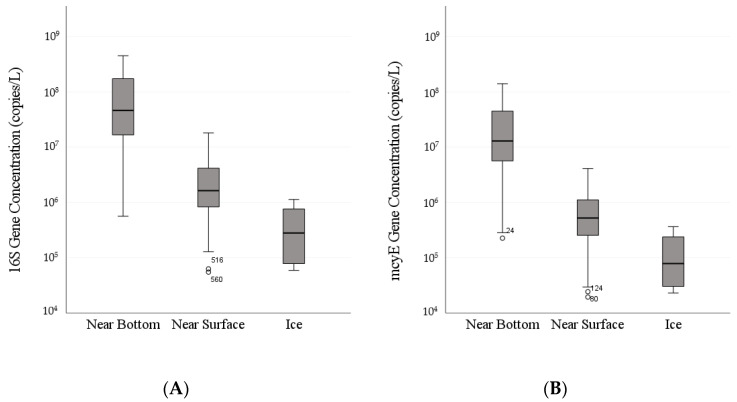
Concentrations of *M. aeruginosa* 16S rRNA (**A**) and *mcyE* (**B**) gene copies in the near-bottom, near-surface, and ice cover from 7 February to 12 March 2019, at the Gilmour Point, Dog Lake field site. Boxes represent the first to third quartile while whiskers represent 95% confidence intervals. Lines within boxes represent the median. Dots represent outliers.

Concentration of the *Microcystis* 16S rRNA gene ranged from 5.62 × 10^5^ to 4.53 × 10^8^ copies per liter of water sampled near the bottom of the water column, with a mean of 9.99 × 10^7^ copies per liter. In samples near the top of the water column, concentrations of the 16S rRNA gene ranged from 5.49 ×10^4^ to 1.81 × 10^7^ copies per liter, with a mean of 3.29 × 10^6^ copies per liter. 16S rRNA concentrations in ice cover samples ranged from 5.90 × 10^4^ to 1.14 × 10^6^ copies per liter, with a mean of 4.34 × 10^5^ copies per liter. There was a statistically significant difference between 16S rRNA gene concentrations in the ice cover, near-surface, and near-bottom (ANOVA: F(147) = 140.884, *p* < 0.001). The assumption of homogeneity of variances was not violated. Post hoc analysis with Tukey’s HSD indicated that 16S rRNA concentration in the near-bottom was significantly higher than in both the near-surface (*p* < 0.001) and ice cover (*p* < 0.001), and 16S rRNA concentration in the near-surface was higher than in the ice cover (*p* < 0.001) (Figure 3A).

The concentration of the *Microcystis mcyE* gene ranged from 2.26 × 10^5^ to 1.41 × 10^8^ copies per liter of water sampled near the bottom of the water column, with a mean of 2.93 × 10^7^ copies per liter. In near-surface samples, the concentration of the *mcyE* gene ranged from 1.94 × 10^4^ to 4.11 × 10^6^ copies per liter, with a mean of 8.49 × 10^5^ copies per liter. *mcyE* concentrations in ice cover samples ranged from 2.30 × 10^4^ to 3.65 × 10^5^ copies per liter, with a mean of 1.29 × 10^5^ copies per liter. There was a statistically significant difference between *mcyE* gene concentrations in the ice cover, near-surface, and near-bottom (ANOVA: F(147) = 144.517, *p* < 0.001). The assumption of homogeneity of variances was not violated. Post hoc analysis with Tukey’s HSD indicated that *mcyE* concentration in the near-bottom was significantly higher than in both the near-surface (*p* < 0.001) and in ice cover samples (*p* < 0.001), and *mcyE* concentration in the near-surface was higher than in the ice cover (*p* < 0.001) (Figure 3B).

Vertical distribution of the overwintering pelagic *Microcystis* population was influenced by both the main effects (sampling time, water column level, and depth), and the interaction effects (sampling time × depth, and pelagic level × depth; Table 2). Based on our measure of effect strengths (partial*η*_p_^2^), the interaction effect between “water column level × depth” had an F-value an order of magnitude greater than the other significant interaction term (i.e., sampling time × depth).

**Figure 4:**
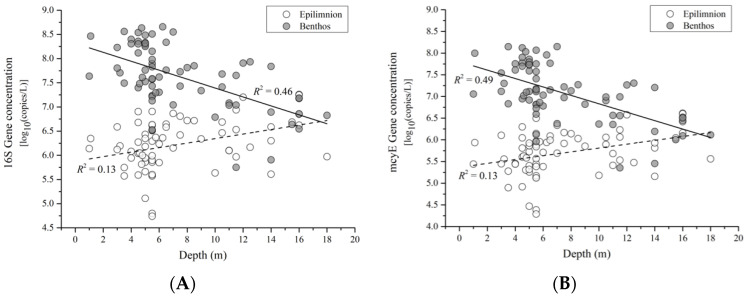
Depth-dependent distribution of overwintering *Microcystis* populations. Visualization of significant interaction effects from the GLMs using concentrations of *M. aeruginosa* (**A**) 16S rRNA and (**B**) *mcyE* gene copies as the dependent variables. (**A**,**B**): interaction effect between water column level (near-surface and near-bottom) and lakebed depth.

Our post hoc Pearson correlation analysis revealed that there was a significant positive correlation between near-surface *M. aeruginosa* 16S gene concentration and depth (*R*^2^ = 0.13, *p* = 0.002, N = 69), while a significant negative correlation was found between near-bottom *M. aeruginosa* 16S gene concentration and depth (*R*^2^ = 0.46, *p* < 0.001, *n* = 69; Figure 4A). Similar correlation patterns were also found between near-surface *mcyE* gene concentration and depth (positive correlation, *R*^2^ = 0.13, *p* = 0.002, *n* = 69) and near-bottom *mcyE* gene concentration and depth (negative correlation, *R*^2^ = 0.49, *p* < 0.001, *n* = 69; Figure 4B).

**Figure 5:**
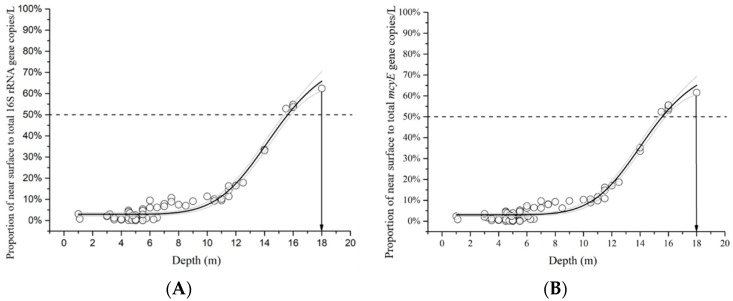
Ratio of near-surface to total *M. aeruginosa* 16S rRNA (A) and *mcyE* (**B**) concentration in gene copies per liter plotted against lakebed depth in meters near the Gilmour Point, Dog Lake field site. Ratio was calculated using raw data of concentrations of *M. aeruginosa* (**A**) 16S rRNA and (**B**) *mcyE* gene concentrations (copies/L) at each sampling site. Dark solid line shows best logistic fitting and its 95% confidence intervals (gray solid lines). *R*^2^_16S_ = 0.97, *R*^2^*_mcyE_* = 0.98. Graphed log_10_ transformed data is on Supplementary Figure S1.

To visualize the depth dependent distribution pattern of pelagic *Microcystis*, we calculated the ratio of near-surface to near-bottom *M. aeruginosa* using raw data of concentrations of *M. aeruginosa* gene concentrations (copies/L) at each sampling site and plotted against depth. The logistic fitting curve showed that the ratio rose with increased water depth, from roughly 0% in the shallow region to approximately 60% when depth reached 18 m (*R*^2^_16S_ = 0.97, *R*^2^_mcyE_ = 0.98; Figure 5). The logistic fitting curves were generated with the following equation, and coefficients can be found in Table 3.

y=A2+(A1−A2)(1+(xx0)p

**Figure 6:**
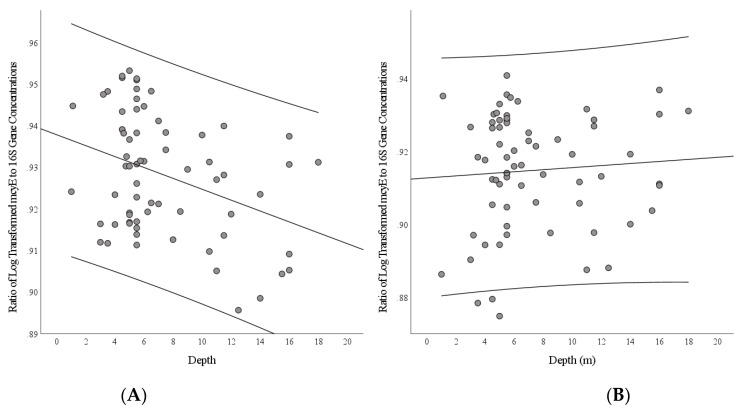
Ratio of the log_10_ of *M. aeruginosa mcyE* and 16S rRNA concentration plotted against depth in meters in the near-bottom (**A**) and near-surface (**B**) at Gilmour Point, Dog Lake. Curved lines represent 95% confidence intervals.

Finally, we created a simple linear regression model that predicted the ratio of the log_10_ transformed *mcyE* gene to log_10_ transformed 16S rRNA gene in the near-bottom. Lakebed depth significantly predicted the *mcyE* to 16S rRNA gene ratio with a negative coefficient, β = −0.359, F(66) = 9.758, *p* = 0.003, *R*^2^ = 0.129 (Figure 6A). We also did this for the near-surface. Lakebed depth did not significantly predict the transformed *mcyE* to 16S rRNA gene concentration near the top of the water column, β = 0.071, F(66) = 0.335, *p* = 0.564, *R*^2^ = 0.005 (Figure 6B).

We plotted residuals for all regressions and found that patterns of the residuals were random, supporting the choice of linear regressions.

## 4. Discussion

In this study, we quantified the relative concentrations of the 16S rRNA and the *mcyE* genes from a prominent bloom forming cyanobacteria species, *M. aeruginosa*, near the lake surface and bottom at different depths in a temperate dimictic lake in Southern Ontario during the winter. We found *Microcystis* present near the top of the pelagic column, near the bottom of the pelagic column, and ice cover of Dog Lake. Near-bottom concentrations were as high as 4.5 × 10^8^ 16S rRNA gene copies per liter of water sampled, which is in line with the literature [36,52], indicating that it was a potentially important source of inoculum in Dog Lake. However, concentrations in the near-surface and ice cover were also high, reaching 1 × 10^6^ cells per liter of water sampled in some areas of the lake. This was comparable to *Microcystis* winter cell densities in Lake Taihu, China, during a persistent bloom that was visible through satellite imaging [53], indicating that they are also potential sources of bloom inoculum. The winter surface and ice cover may be particularly significant sources of potential inoculum in deeper areas of lakes previously thought to have low potential for nascent CHABs. Our findings were similar to previous work on overwintering *Microcystis*, which emphasized the dominant role of benthic inoculum [27,52,54,55], with a small pelagic overwintering population [56]. As our eDNA sampling method used filters with a pore size of 1.2 μm and *Microcystis* cells measure 2–8 μm in diameter, we primarily captured live cells and demonstrated that living, viable cyanobacteria was present in those areas.

Interest in the use of qPCR, and other molecular tools on environmental DNA samples for detecting, characterizing and quantifying cyanobacterial blooms has surged in the past two decades [57,58]. Work on developing qPCR protocols for detecting toxic cyanobacterial strains have found high correlation between the presence of the *mcy* gene family and microcystins [59]. qPCR has also been used for the quantification of environmental bacterial samples and have corresponded with high precision to absolute cell numbers [60]. Otten et al. [36] used the *Microcystis mcyE* and *cpcA* genes to quantify the presence of toxigenic and total *Microcystis* in Lake Taihu, China, and found significant positive correlations between microcystin concentration and concentrations of both genes. They also examined the ratio of the *mcyE* gene to the *cpcA* gene as a measure of toxigenicity, finding highly variable ratios at different areas of the lake. Further work by Chiu et al. [60] found similarly high correlations between qPCR results on eDNA with cyanotoxins with a multiplexed qPCR approach that simultaneously quantified genes from two common CHAB genera, *Microcystis* and *Cylindrospermopsis*. Although more work in correlating cellular biomass and eDNA concentrations in specific conditions is needed to use eDNA as an absolute measure of cyanobacteria abundance, relative abundance can be estimated with similar conditions between sampling sites.

We found higher copy numbers of both the 16S rRNA and *mcyE* genes in areas of the lake with shallower lakebeds. Samples from the near-bottom had higher eDNA concentrations overall. This was supported by the results of Cao et al. [61] and Liu et al. [62], who found that vegetative growth and subsequent recruitment of overwintering *M. aeruginosa* in Lake Taihu, China was mediated by cumulative temperature. Lab experiments indicated that growth renewed between 5 and 9 °C, and recruitment began at 14 °C. Similarly, Thomas and Walsby [63] found that *Microcystis* in dark and low temperature conditions had low buoyancy recovery after autumnal decline due to reduced rates of protein and gas vesicle synthesis. Therefore, it is likely that lake bottom *Microcystis* in shallower areas begin growth and recruitment earlier than in deeper areas that have less light availability and are slower to warm. Therefore, near-bottom *Microcystis* in shallower areas of the lake likely have a competitive advantage over lake-bottom *Microcystis* in deeper areas as sources of inoculum.

Not only does the lake bottom in deeper lakebed areas receive fewer the environmental cues that promote active buoyancy recovery and recruitment, the passive processes that mediate recruitment may also favor the lake bottom of shallow lakebed areas. Stahl–Delbanco and Hansson [64] found that the recruitment rate of *Microcystis* increased in the presence of benthic macrofauna, indicating that bioturbation may also be a contributor to resuspension. Their findings suggested that recruitment rates may be higher in shallower littoral areas where benthic invertebrates such as *Asellus aquaticus* (Isopoda) dominate, compared to deeper areas dominated by pelagic invertebrates that cause less bioturbation, such as chironomids. Abiotic hydrological effects may also favor cyanobacterial recruitment from the near-bottom of shallow lakebed areas. Wind-induced mixing typically occurred through Langmuir circulation and was only significant in shallow lakebeds, up to a depth of 4–6 m [65,66]. Verspagen et al. [30] found that benthic colonies of *Microcystis* did not have sufficient carbohydrate content to restore buoyancy during spring recruitment, and buoyancy state was instead largely the result of mixing and subsequent resuspension. Therefore, benthic recruitment may be primarily driven by processes such as wind-induced mixing and heavy precipitation events, which most strongly mix shallower areas of lakes.

Water column distribution of *Microcystis* depended on lake depth. As the depth of the lakebed increased, we observed a shift from lake bottom to top *Microcystis* dominance in Dog Lake. The persistence of planktonic, vegetative *Microcystis* in low temperatures is supported by Ma et al. [53], who found that blooms persisted in Lake Taihu, China over the winter at temperatures below 10 °C. Although Lake Taihu is subtropical and does not experience ice cover, overwintering vegetative cells in laboratory conditions maintained low levels of photosynthetic activity at temperatures as low as 2 °C, and low temperatures decreased loss rate. As water temperatures under ice cover in lakes in Southern Ontario can be as high as 4 °C, this supports our findings on the persistence of overwintering pelagic planktonic populations and their potential as sources of inoculum. Overwintering *Microcystis* in other temperate, dimictic lakes with winter ice cover may also be primarily surface dominated close to the center of lake basins, and sources of inoculum depend on the hydrological and topographic characteristics of lakes.

Although lake bottom populations of *Microcystis* predominated overall, cell density in different parts of the water column may not be indicative of their actual contribution to blooms. Verspagen et al. [29] examined coupling between benthic and pelagic populations of *Microcystis*. They found that sediment recruitment occurred throughout the year and was counterbalanced by high sedimentation rates. Additionally, their findings suggested that there was horizontal transportation of sedimented *Microcystis* from shallow to deeper areas of the lake. Although the pelagic *Microcystis* population was much smaller in the spring, their model implicated it as a more significant contributor to summer blooms, indicating that controlling it may be key to effective management.

In addition to the near-surface and near-bottom, we also found concentrations of *Microcystis* in the ice cover. This was similar to the results of Vasas et al. [32], who found populations of *Microcystis viridis* with high viability from a visible bloom frozen in ice cover. However, our study found *Microcystis* in ice cover from a wider area in a natural lake, and compared it with lake surface and bottom sources as well, further demonstrating the potential of ice cover as a previously unexpected inoculum source. Our results are supported by Park [34], who found that *Microcystis*, *Anabaena*, *Oscillatoria,* and *Aphanizomenon* in both colonial and planktonic form had high viability after cryopreservation at −60 °C without a cryoprotectant after as long as two years. This indicates that these CHAB species in general are probably highly resistant to freezing, an inference supported by the lack of a spore or akinete form in these particular species. It is possible that the extracellular lipopolysaccharide/polysaccharide mucilage of colonial cyanobacteria has a cryoprotectant effect, which is common in other mucilage forming bacteria [67].

We observed a weak but significant negative correlation between the ratio of *M. aeruginosa mcyE* to 16S rRNA gene concentration to lakebed depth in the near-bottom, indicating potentially higher toxicity in near-bottom *Microcystis* overwintering close to the shoreline and in other shallower areas. However, no significant relationship was present in the top of the water column. As there was no significant difference between the *mcyE* to 16S rRNA ratio in the near-surface and the *mcyE* to 16S rRNA ratio in the near-bottom across all lakebed depths, it is unlikely that this was solely the result of difference in water column depth. This variation in genotypes was similar to the results of Otten et al. [36], who found a mean *mcyE* to *cpcA* ratio of 36% ± 12% in the *Microcystis* of Lake Taihu, which was similar to the mean *mcyE* to 16S rRNA ratio of 30% ± 7% in Dog Lake over the previous 3 years [58]. This negative correlation may be driven by differences in light intensity on the lake bottom between shallower and deeper areas of the lake. The toxicity of *Microcystis* has been positively correlated with light and temperature [68,69,70]. Photooxidative damage from high irradiance upregulates the microcystin pathway due to its protective effect against reactive oxygen species [71].

Although our study quantified and compared overwintering *Microcystis* populations in different parts of the water column, we did not examine their relative contributions as sources of inoculum to summer blooms. Additional research is needed to determine rates of reproduction and photosynthetic activity of CHAB species at different areas of water bodies, particularly in the top of the water column of lakes during and after spring recruitment. The hydrological characteristics of temperate, shallow lakes, and therefore the mechanisms that influence recruitment remain poorly understood, particularly in terms of interactions between macrophytes and other biotic influences with stratification and mixing [72]. The impact of longer timescale shifts in temperature and N:P ratios may also impact bloom formation and composition [73]. Furthermore, this study was specific to *Microcystis* through quantification of the *M. aeruginosa* 16S rRNA and *mcyE* genes. Perakis et al. [72] found that summer planktonic *Microcystis* populations did not significantly increase in response to high inputs from benthic recruitment, while *Anabaena*, *Aphanizomenon*, and *Coelosphaerium* did, indicating that different sources of inoculum may be dominant between different CHAB genera. Therefore, other CHAB species, particularly filamentous genera that lack mucilage and gas vesicles, such as *Anabaena* and *Aphanizomenon*, may differ in their overwintering distributions.

## 5. Conclusions

The results of our study have several implications for bloom monitoring and control strategies and for future. Near-surface and ice cover populations of *Microcystis* and other bloom-forming species may compose a greater portion of the overwintering cyanobacterial population than previously thought, particularly in deeper areas of lakes, and may be significant contributors of inoculum in the spring. The water column distribution of overwintering cyanobacteria may be dependent on lakebed depth. Efforts in early CHAB modeling should consider the top of the water column and ice cover of lakes in the spring, in addition to lake-bottom, sedimentary, and fluvial-inflow sources of inoculum.

## Figures and Tables

**Table 1:** Primers used for qPCR to amplify fragments of the *Microcystis* 16S rRNA gene and *mcyE* gene.

Target	Primer	Sequence (5′ to 3′)	Size	Reference
*Microcystis* 16S gene	*MIC* 16S-F	GCCGCRAGGTGAAAMCTAA	220 bp	[50]
	*MIC* 16S-R	AATCCAAARACCTTCCTCCC		
*Microcystis mcyE* gene	*MIC mcyE*-F	AAGCAAACTGCTCCCGGTATC	120 bp	[51]
	*MIC mcyE*-R	CAATGGGAGCATAACGAGTCAA		

**Table 2:** Results of a univariate GLM using concentrations of the *M. aeruginosa* (A) 16S rRNA and (B) *mcyE* gene as the dependent variable. Df refers to degrees of freedom, F refers to the ratio of population variances, and P indicates significance with an alpha threshold of 0.05.

Factor	Df	F	P	Wilks’ Partial *η*_p_^2^
**(A) Concentrations of *M. aeruginosa* 16S rRNA gene**				
Sampling time	5	6.25	<0.001	0.201
Water column level (near-surface, near-bottom, bottom)	1	308.12	<0.001	0.713
Depth	1	9.70	=0.002	0.073
Sampling time × depth	5	5.66	<0.001	0.186
Water column level × depth	1	81.63	<0.001	0.397
Error	124			
**(B) Concentrations of *M. aeruginosa mcyE* gene**				
Sampling time	5	7.48	<0.001	0.232
Water column level (near-surface, near-bottom)	1	323.39	<0.001	0.723
Depth	1	10.67	=0.001	0.079
Sampling time × depth	5	6.15	<0.001	0.199
Water column level × depth	1	86.54	<0.001	0.411
Error	124			

Interaction terms were excluded from the final model if *p* > 0.05 starting with the highest-level interaction term, but the next hierarchical level of interactions was retained if one term had *p* < 0.05. (Note that further exclusion of single interaction terms did not alter the results qualitatively).

**Table 3:** Coefficients for GLM equation predicting ratio of near-surface/near-bottom gene concentrations with depth.

Gene	Coefficient	Value	Std. Error
16S rRNA	A_1_	0.02924	0.00382
	A_2_	0.80606	0.07893
	X_0_	14.7098	0.49923
	p	7.19072	0.69382
*mcyE*	A_1_	0.03037	0.00365
	A_2_	0.77372	0.06673
	X_0_	14.4735	0.43581
	p	7.41774	0.68911

## Data Availability

The data presented in this study are available in Supplementary Materials.

## Supplementary Materials

The following are available online at https://www.mdpi.com/article/10.3390/microorganisms9081718/s1, Figure S1: Sampling scheme in this study by date, Table S1: Ratio of log_10_ transformed *M. aeruginosa* 16S rRNA and *mcyE* concentrations in the near surface and near bottom plotted against depth.
