# Supplementary material for: Depth-Dependent Spatiotemporal Dynamics of Overwintering Pelagic Microcystis in a Temperate Water Body
Source: Microorganisms. 2021 Aug 12;9(8):1718. doi: 10.3390/microorganisms9081718 (PMC8399979; doi:10.3390/microorganisms9081718)
Supplement: Supplementary file 1 [file microorganisms-09-01718-s001.zip › SI Tables and Figures.pdf]

Depth-Dependent Spatiotemporal Dynamics of Overwintering Pelagic *Microcystis* in a Temperate Water Body Supplementary Information

Haolun Tian [1,†], Junjie Jin [1,†], Bojian Chen [2,†], Daniel D. Lefebvre [1], Stephen C. Lougheed [1,2] and Yuxiang Wang [1,2,*]

6. Supplementary Information

SI Table S1. Sampling scheme in this study by date

Sampling Date	# of Surface Samples	# of Bottom Samples	# of Ice Samples
2019-02-07	10	10	0
2019-02-14	10	10	0
2019-02-21	10	10	0
2019-02-28	14	14	15
2019-03-07	15	15	0
2019-03-12	10	10	0

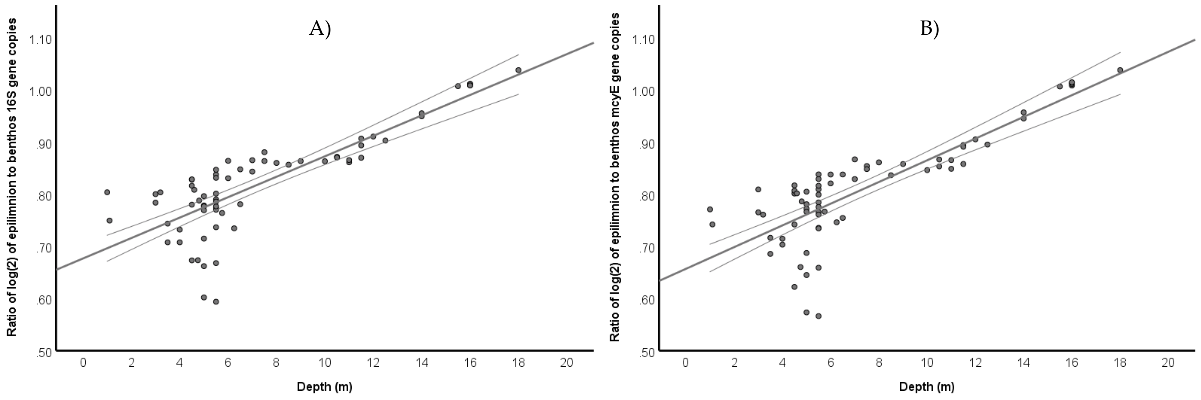

SI Figure S1. Ratio of log10 transformed *M. aeruginosa* 16S rRNA (**A**) and *mcyE* (**B**) concentrations in the near surface and near bottom plotted against depth in metres near the Gilmour Point, Dog Lake field site. Curved lines represent 95% confidence intervals.
